# Eurasian jays (*Garrulus glandarius*) conceal caches from onlookers

**DOI:** 10.1007/s10071-014-0743-2

**Published:** 2014-03-18

**Authors:** Edward W. Legg, Nicola S. Clayton

**Affiliations:** Department of Psychology, University of Cambridge, Downing Street, Cambridge, CB2 3EB UK

**Keywords:** Eurasian jay, Cache protection, Caching, Corvids, Social cognition

## Abstract

Animals that cache food risk having their stored food pilfered by conspecifics. Previous research has shown that a number of food-caching species of corvid use strategies that decrease the probability of conspecifics pilfering their caches. In this experiment, we investigated whether Eurasian jays (*Garrulus glandarius*) would choose between caching behind an opaque and caching behind a transparent barrier whilst being observed by a conspecific. If caching in out-of-sight locations is a strategy to prevent conspecifics from pilfering these caches, then the jays should place a greater proportion of caches behind the opaque barrier when being observed than when caching in private. In accordance with this prediction, jays cached a greater proportion of food behind the opaque barrier when they were observed than when they cached in private. These results suggest that Eurasian jays may opt to cache in out-of-view locations to reduce the likelihood of conspecifics pilfering their caches.

**Electronic supplementary material:**

The online version of this article (doi:10.1007/s10071-014-0743-2) contains supplementary material, which is available to authorized users.

## Introduction

Food-caching corvids, such as Eurasian jays (*Garrulus glandarius*), store food in order to consume it at a future time. Many corvid species possess observational spatial memory (e.g. Bednekoff and Balda 1996; Bugnyar and Kotrschal 2002; Watanabe and Clayton 2007) which allows them to remember and locate the caches they have seen a conspecific make. Thus, caches made by corvids are particularly susceptible to being pilfered by conspecifics. Consequently, corvids are known to exhibit a range of strategies that protect their caches from being pilfered by conspecifics.

Eurasian jays have been shown to use certain cache protection strategies such as caching in quieter locations when a conspecific is within earshot (Shaw and Clayton 2013). Anecdotal evidence suggests that wild Eurasian jays preferentially cache near bushes or trees (Chettleburgh 1952). These landmarks may aid the jays’ ability to relocate caches (Bennett 1993) and can also prevent conspecifics observing caching episodes. Experiments have shown that both ravens (*Corvus corax)* and western scrub-jays (*Aphelocoma californica*) use similar locations as part of their cache protection repertoire and specifically show a preference to cache behind barriers in the presence of conspecifics as opposed to when they cache alone (Bugnyar and Kotrschal 2002; Dally et al. 2005). If Eurasian jays’ preference to cache near vertical landmarks is a cache protection tactic, then they should cache in out-of-sight locations when conspecifics are present, but not when they cache in private.

To test this hypothesis, we provided Eurasian jays with two cache locations: one *in*-*view* and one *out*-*of*-*view* and varied whether the jays cached whilst they were observed or in private. If jays use out-of-view locations to protect their caches, then they are expected to show a stronger preference for caching in these out-of-sight locations whilst a conspecific is observing than when caching in private.

## Methods

### Subjects

We tested eight Eurasian jays (four females and four males, aged 6 years). They were housed in a large outdoor aviary (20 × 6 × 3 m) and tested in indoor compartments (2 × 1 × 2 m) that they accessed from the aviary through a trap window. They were fed on a maintenance diet of soaked dog biscuits, cheese, seeds, nuts and fruit and had ad libitum access to water. The maintenance diet was removed approximately 2 h before testing began and was returned after the caching phase of the experiment.

### Apparatus

We used two adjacent indoor compartments (3 × 1 × 2 m), one for the cacher and, if applicable, one for the observer. The cacher’s compartment contained a platform at 1 m above ground. A transparent window (30 cm by 55 cm) was positioned between the compartments.

**Fig. 1:**
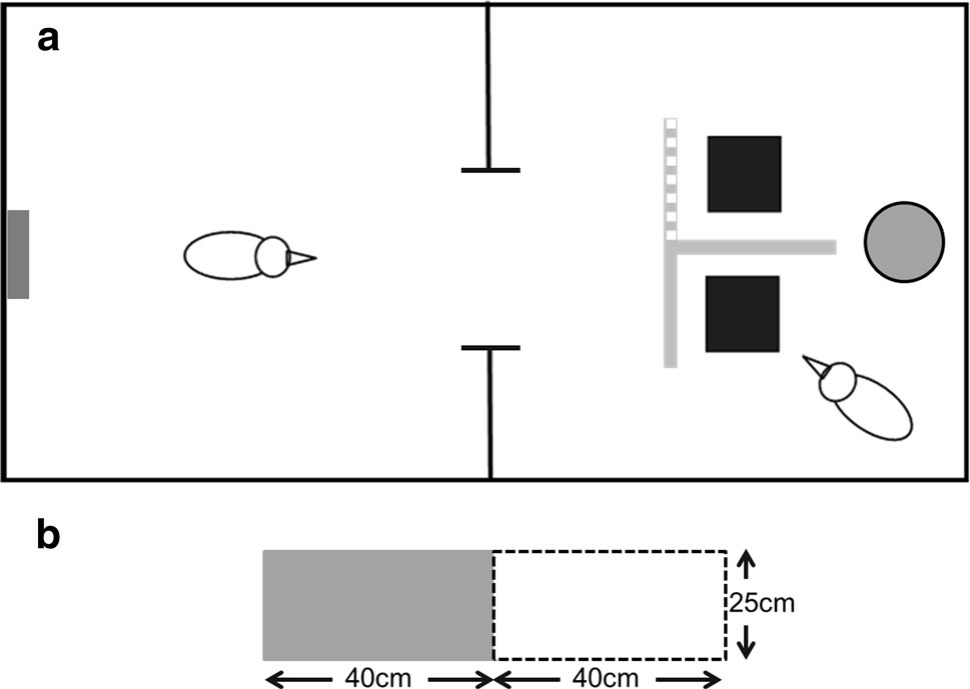
**a** Schematics of the two compartments. The *left* compartment is the observer’s compartment; the *grey bar* to the far *left* of the compartment indicates the position of the perch. The *right* compartment is the cacher’s compartment. The *T*-shape represents the *T*-shaped barrier, the *grey* and *white* hatched arm represents the transparent arm, the *solid grey* arm and stem are opaque. The *two black squares* are the caching trays and the *grey circle* is the bowl containing 30 peanut halves. **b** The *T*-shaped barrier as seen from the observer’s perspective. The *black and* *white outlined area* represents the transparent arm, the *solid grey area* represents the opaque arm

A ‘*T*-shaped’ barrier (see Fig. 1; three 25 cm × 40 cm sheets forming two arms and a stem) was placed in the centre of the platform in the cacher’s compartment to create an *in*-*view* and an *out*-*of*-*view* cache locations. The stem and one arm of the ‘*T*’ were made of opaque plastic (*out*-*of*-*view*) and the second arm was made from transparent Perspex© (*in*-*view*). The barrier was 25-cm high, which hid the caching tray from the observer’s view but did not completely hide the cacher.

Two rectangular seedling trays (3 × 3 pots filled with sand) were placed behind the ‘*T*-shaped’ barrier such that one tray was behind the Perspex (*in*-*view*) and the other was behind the opaque plastic (*out*-*of*-*view)*. The Eurasian jays tested were familiar with caching in similar seedling trays, and by using sand as the substrate, we prevented observers from hearing the caching events (Shaw and Clayton 2013).

### Procedure

Eurasian jays were tested whilst observed by a conspecific or in private. The presence of a dominant individual leads to subordinates suppressing the amount they cache (Shaw and Clayton 2012), thus to control for the influence of dominance, where possible, the birds were observed by both a subordinate and a dominant bird leading to 3 trial types with the *observed by subordinate* and *observed by dominant* trials being collapsed into an *observed* condition.

Jays were initially tested with the ‘*T*-shaped’ barrier in one orientation. Subsequently, the three conditions were repeated with the ‘*T*-shaped’ barrier in the opposite orientation. The order in which the jays experienced the two orientations (*Orientation A*: *out*-*of*-*view* location to the right of the barriers stem; *Orientation B*: *out*-*of*-*view* location to the left of barriers stem) was counterbalanced across birds.

A bowl containing 30 peanut halves was placed close to the stem of the *T*-shaped barrier and equidistant to the two caching locations. After 15 min, the birds were released back into the aviary and the caching tray and bowl of peanuts were removed (the *T*-shaped barrier remained in place). The experimenter counted the number of caches made in the two locations by emptying each of the individual pots of sand. Any caches were re-hidden in their original location.

After approximately 2.5 h, the cacher received a 15-min-long recovery session, always run in private, in which they had access to both trays and were able to recover their caches. The cachers received 1 trial and 1 recovery session each day. After the 15-min recovery session, the bird was released and the two trays were removed from the compartment. Any remaining caches were counted by the experimenter and their locations noted. Any items in new locations were scored as re-cached. Items that were absent were scored as retrieved.

Three birds (1 male and 2 female) served as subordinate observers (Adlington, Ainslie, Purchas) and three birds (2 male and 1 female) served as dominant observers (Wilson, Hoy, Ohu). Birds were tested as cachers before they subsequently served as observers. This was done to eliminate the possibility that the birds’ caching behaviour could be influenced by their experience of being an observer. However, this was not possible for all birds, such that one bird (Adlington) was tested as a cacher after she had served as an observer. Like western scrub-jays (Grodzinski et al. 2012), all observers appeared to show an interest in the behaviour of the cacher.

### Analysis

For each trial, we calculated the proportion of items cached in the *out*-*of*-*view* location and the proportion of items retrieved out of all items cached during the recovery session for both *in*-*view* and *out*-*of*-*view* locations. The data for the *observed* condition were calculated by taking the mean value from the *observed by subordinate* and the *observed by dominant* condition.

Data were analysed using R 3.0.0. Due to the small sample size, data were analysed using permutation tests. Permutation tests obtain their test statistic by calculating all possible values by re-arranging the labels on the observed data points (2^*N*^ permutations are run; *N* is the number of paired data points). Thus, they are a subset of nonparametric tests that do not make an assumption about the distribution of the data (Anderson 2001). Unless otherwise stated, all tests were non-directional (two-tailed *p* values). Alpha was set at 0.05.

## Results

### Caching

Two birds did not habituate to the compartment containing the ‘*T*-shaped’ barrier after 3 months of experience and thus could not be tested. The most dominant bird, Wilson, could not be tested in the *observed by dominant* condition, because no bird was dominant to him.

**Fig. 2:**
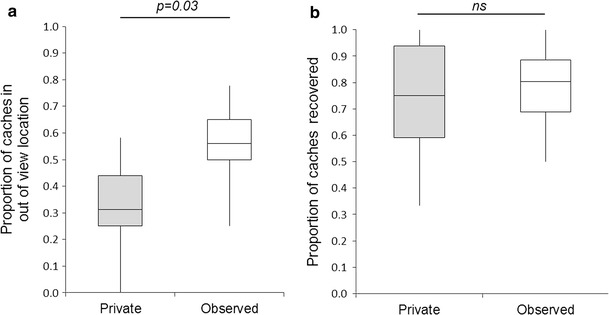
**a** The proportion of caches made in the out-of-view location. **b** The proportion of caches retrieved. The *boxes* show the median and interquartile range, the *whiskers* represent the maximum and minimum values

The Eurasian jays cached a median of two and a half items (IQR = 1.21). The number of items cached did not vary between the two orientations (*n* = 6, *Z* = 0.55, *p* = 0.625). The birds cached a similar number of items in both the *observed* and the *private* conditions (*n* = 6, *Z* = −0.38, *p* = 0.625). However, the birds changed their preferred caching location between the two conditions by caching a greater proportion of items in the *out*-*of*-*view* tray whilst observed than when in the *private* condition (*n* = 6, *Z* = −2.00, *p*_one-tailed_ = 0.03; see Fig. 2).

### Recovery

Only two birds re-cached items in novel locations (Adlington in an *observed by dominant* trial and Hunter in a *private* trial) such that no analysis could be performed regarding the jays’ re-caching behaviour.

The Eurasian jays retrieved a median of 1.67 items (IQR = 0.83) which accounted for 72.9 % of their caches. They retrieved a similar proportion of items in the *private* and the *observed* conditions (*n* = 6, *Z* = 1.07, *p* = 0.19). This pattern was observed both for the *in*-*view* (*n* = 6, *Z* = −1.64, *p* = 0.13) and the *out*-*of*-*view* locations (*n* = 6, *Z* = −1.40, *p* = 0.25).

## Discussion

Eurasian jays preferred to cache in out-of-view locations when they were observed by a conspecific but not when they were caching in private. However, behaviour at recovery was not influenced by whether the jays had been observed during caching. Thus, the Eurasian jays’ choice of cache locations was influenced by social context. The jays’ preference for caching in the *out*-*of*-*view* location whilst being observed may be an attempt to alleviate the threat that conspecifics pose to a cache. By caching a greater proportion of peanuts in out-of-view locations, the Eurasian jays are able to limit the conspecific observer’s visual access to the exact location of the caches. This may reduce the probability of caches being found and pilfered by the observer.

Importantly, the only experimental manipulation to the two cache locations was whether they were *in*-*view* or *out*-*of*-*view*. This meant our procedure was similar to the one used by Dally et al. (2005) to test western scrub-jays’ preference for caching in out-of-view locations. Both caching locations were equidistant to the observer’s compartment as distance influences the caching locations used by western scrub-jays when observed (Dally et al. 2005). Moreover, birds always received intact caches at recovery, because cache loss at recovery can influence their choice of cache location (de Kort et al. 2007). The barrier used by Dally et al. (2005) to create the *out*-*of*-*view* caching location was the full height of the cage, which meant both that the observer did not see the caching location and that the cacher could not see the observer whilst caching. This means that the cacher may have simply spent more time out of the observer’s sight—regardless of whether they were caching. However, Dally et al. (2005) found that the western scrub-jays did not prefer to spend more time behind a particular barrier; thus, the preference for the out-of-view location was specific to caching. In the current study, we used a 25-cm-high barrier, which was 5 cm shorter than the window between compartments. This meant that the observer could not see the *out*-*of*-view caching location but that the cacher could see the observer from both the *in*-*view* and *out*-*of*-*view* caching locations. Thus, the Eurasian jays’ preference for the *out*-*of*-*view* location cannot be explained by a general propensity to not see conspecifics before caching. Instead, the jay’s choice of caching location appears to be dependent on what conspecifics can or cannot see.

The Eurasian jays’ preference for caching in locations that an observer cannot see adds to previous research that showed that these birds can account for what conspecifics can hear (Shaw and Clayton 2013). The present findings raise the possibility that Eurasian jays may possess a similarly rich repertoire of cache protection strategies to other corvids. Future experiments may establish the range of Eurasian jays’ cache protection strategies and the underlying mechanism.

## Electronic supplementary material

Below is the link to the electronic supplementary material.

Supplementary material 1 (DOCX 39 kb)
