# Supplementary material for: Eurasian jays (Garrulus glandarius) conceal caches from onlookers
Source: Anim Cogn. 2014 Mar 18;17(5):1223–6. doi: 10.1007/s10071-014-0743-2 (PMC4138428; doi:10.1007/s10071-014-0743-2)
Supplement: Supplementary file 1 — Supplementary material 1 (DOCX 39 kb) [file 10071_2014_743_MOESM1_ESM.docx]

**Supplementary Material**

**Table 1**: Raw data for caches and recovery

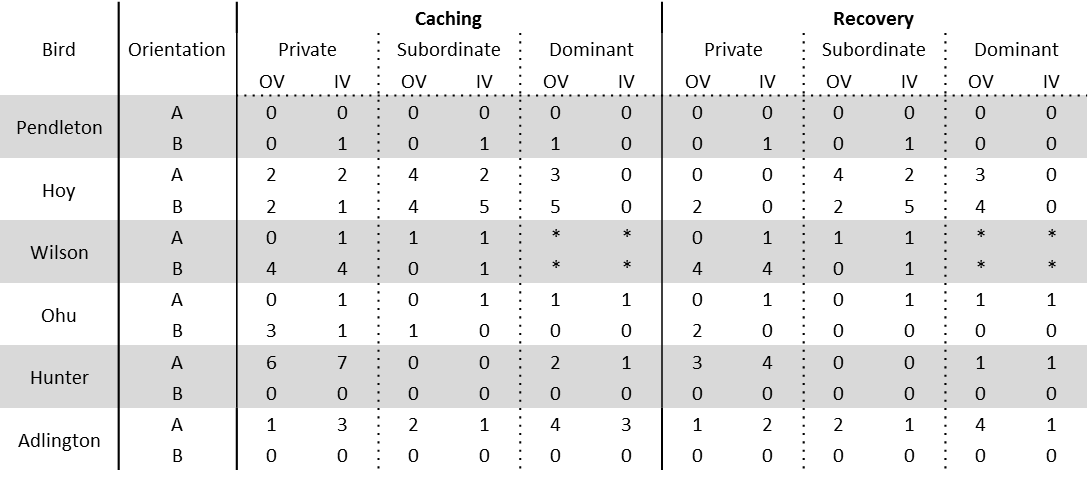


*The column ‘Orientation’ refers to the orientation of the T-shaped barrier. A = opaque to right, B = opaque to left. ‘IV’ =in view location; ‘OV’ = Out of view location. The private column refers to data from the private condition, the ‘Subordinate’ column refers to data while the observer was subordinate and the ‘Dominant’ column refers to data while the observer was dominant.’*’ indicate conditions that could not be run on those birds.*
